# Lung response to prone positioning in mechanically-ventilated patients with COVID-19

**DOI:** 10.1186/s13054-022-03996-0

**Published:** 2022-05-07

**Authors:** Alessandro Protti, Alessandro Santini, Francesca Pennati, Chiara Chiurazzi, Michele Ferrari, Giacomo E. Iapichino, Luca Carenzo, Francesca Dalla Corte, Ezio Lanza, Nicolò Martinetti, Andrea Aliverti, Maurizio Cecconi

**Affiliations:** 1 Department of Biomedical Sciences, Humanitas University, Pieve Emanuele, Milan, Italy; 2 Department of Anesthesia and Intensive Care Units, IRCCS Humanitas Research Hospital, Rozzano, Milan, Italy; 3 Dipartimento di Elettronica, Informazione e Bioingegneria, Politecnico di Milano, Milan, Italy; 4 Department of Radiology, IRCCS Humanitas Research Hospital, Rozzano, Milan, Italy

**Keywords:** Acute respiratory distress syndrome, Coronavirus disease 2019, Hypoxia, Mechanical ventilation, Pneumonia, Prone positioning

## Abstract

**Background:**

Prone positioning improves survival in moderate-to-severe acute respiratory distress syndrome (ARDS) unrelated to the novel coronavirus disease (COVID-19). This benefit is probably mediated by a decrease in alveolar collapse and hyperinflation and a more homogeneous distribution of lung aeration, with fewer harms from mechanical ventilation. In this preliminary physiological study we aimed to verify whether prone positioning causes analogue changes in lung aeration in COVID-19. A positive result would support prone positioning even in this other population.

**Methods:**

Fifteen mechanically-ventilated patients with COVID-19 underwent a lung computed tomography in the supine and prone position with a constant positive end-expiratory pressure (PEEP) within three days of endotracheal intubation. Using quantitative analysis, we measured the volume of the non-aerated, poorly-aerated, well-aerated, and over-aerated compartments and the gas-to-tissue ratio of the ten vertical levels of the lung. In addition, we expressed the heterogeneity of lung aeration with the standardized median absolute deviation of the ten vertical gas-to-tissue ratios, with lower values indicating less heterogeneity.

**Results:**

By the time of the study, PEEP was 12 (10–14) cmH_2_O and the PaO_2_:FiO_2_ 107 (84–173) mmHg in the supine position. With prone positioning, the volume of the non-aerated compartment decreased by 82 (26–147) ml, of the poorly-aerated compartment increased by 82 (53–174) ml, of the normally-aerated compartment did not significantly change, and of the over-aerated compartment decreased by 28 (11–186) ml. In eight (53%) patients, the volume of the over-aerated compartment decreased more than the volume of the non-aerated compartment. The gas-to-tissue ratio of the ten vertical levels of the lung decreased by 0.34 (0.25–0.49) ml/g per level in the supine position and by 0.03 (− 0.11 to 0.14) ml/g in the prone position (*p* < 0.001). The standardized median absolute deviation of the gas-to-tissue ratios of those ten levels decreased in all patients, from 0.55 (0.50–0.71) to 0.20 (0.14–0.27) (*p* < 0.001).

**Conclusions:**

In fifteen patients with COVID-19, prone positioning decreased alveolar collapse, hyperinflation, and homogenized lung aeration. A similar response has been observed in other ARDS, where prone positioning improves outcome. Therefore, our data provide a pathophysiological rationale to support prone positioning even in COVID-19.

**Supplementary Information:**

The online version contains supplementary material available at 10.1186/s13054-022-03996-0.

## Background

Prone positioning was recommended for moderate-to-severe acute respiratory distress syndrome (ARDS) well before the appearance of the novel coronavirus disease (COVID-19) [1]. It improves survival [1, 2] possibly by reopening or “recruiting” the dorsal non-aerated but perfused lung tissue and diminishing ventral hyperinflation [3–6]. As a result, arterial oxygenation almost always increases, and, more important, ventilation becomes more evenly distributed, with fewer harms from mechanical ventilation [2–6]. Prone positioning carries some risks, including transient desaturation, endotracheal tube obstruction or displacement (up to accidental extubation), reduced venous return, need for more sedation, vomiting, loss of venous access, and pressure sores [2, 6]. The advantages of prone positioning are more likely to outweigh these dangers in moderate-to-severe ARDS, i.e. when the risk of secondary lung damage is higher [1, 6, 7].

Soon after its appearance, prone positioning was recommended by international guidelines and experts [8, 9] and widely used [10, 11] even for moderate-to-severe ARDS related to COVID-19. The underlying assumption was that prone positioning is also beneficial in ARDS due to COVID-19. However, whether the latter should be treated as ARDS of other origins remains controversial [9, 12, 13].

This study aimed to verify whether prone positioning decreases alveolar collapse and hyperinflation and homogenizes lung aeration in patients with early ARDS due to COVID-19. We reasoned that a positive response would support prone positioning even in this novel syndrome.

## Methods

Our institutional review board approved this study (Comitato Etico dell’IRCCS Istituto Clinico Humanitas Rozzano; protocol n. 465/20). Informed consent was obtained according to local regulations.

We enrolled fifteen patients with laboratory-confirmed COVID-19 from 1/3/2020 to 09/12/2020. Inclusion criteria were: (1) a diagnosis of ARDS [14]; (2) ongoing invasive mechanical ventilation with deep sedation and neuromuscular blockade; (3) prone positioning prescribed by the attending physician within 3 days of endotracheal intubation. Forty-six patients were excluded because (1) they had already undergone a lung computed tomography (CT) after endotracheal intubation (*n* = 16); (2) they were too unstable for transfer to the radiology unit (*n* = 9); (3) their body weight exceeded 100 kg (*n* = 6); or (4) none of the authors was available for collecting data, due to the exceptional clinical workload at that time (*n* = 15) (Additional file 1: Table S1).

### Lung morphological response

A recruitment manoeuvre was performed at 45 cmH_2_O of end-inspiratory airway pressure to standardize the lung volume history [15]. After that, a static end-expiratory lung CT without contrast was taken with the patient in the supine position as described in reference 16. Positive end-expiratory pressure (PEEP) was the same as in the intensive care unit (ICU) prior to the study and set at the discretion of the attending physician. Patients were then turned prone. After a new recruitment manoeuvre (as above), a second static CT was taken at the same PEEP level. After that, patients were returned to the ICU in the supine position.

#### Global inflation

The total volume, tissue weight, and gas volume of the whole lung and its non-aerated (density above − 100 HU), poorly-aerated (from − 100 to − 500 HU), normally-aerated (from − 500 to − 900 HU), and over-aerated (below − 900 HU) compartments were measured as in reference 15. The premorbid lung weight was estimated from the subjects’ height [17]. The average lung aeration was expressed as the ratio of total gas volume to total tissue weight [3].

### Regional inflation

These same methods were applied to ten equal vertical and horizontal levels forming each CT slice, from the sternum (ventral) to the vertebra (dorsal) and from the apex (cranial) to the base (caudal) of the lung. The regional aeration was computed as the ratio of gas volume to tissue weight of each vertical and horizontal level [3]. The regional lung morphological response to prone positioning was assessed as the degree of heterogeneity of lung aeration along the sterno-vertebral or cranio-caudal axis. This was quantified with the standardized median absolute deviation of the corresponding ten gas-to-tissue ratios within each subject [18]; higher values indicated more heterogeneity.

The hydrostatic pressure (super)imposed on each vertical level was computed as in reference 19.

### Lung functional response

Gas exchange and respiratory system mechanics were measured 20 min after a recruitment manoeuvre (as above), immediately before and 60 min after prone positioning. Physicians were asked to keep the ventilatory settings as constant as reasonable in the two positions. Those preferring to increase the fraction of inspired oxygen (FiO_2_) during prone positioning (up to 100%) to prevent peri-procedural desaturation were invited to do so in advance when the patient was studied in the supine position. PEEP was the same as in the radiology unit.

### Association between morphological and functional responses

We studied the association between the change in the volume of the non-aerated or over-aerated compartment, or the change in heterogeneity of lung aeration, and: (1) those volumes and heterogeneity in supine position; (2) the change in oxygenation, compliance, and PaCO_2_ in response to prone positioning.

### Statistical analysis

The primary outcome of the study was the global lung morphological response to prone positioning, defined as the change in the total volume of the non-aerated (alveolar collapse) and over-aerated (hyperinflation) compartments from supine to prone [16]. Sample size was based on feasibility rather than statistical power considerations.

Data are presented as median (Q1–Q3) or proportion. They were analysed with the Mann–Whitney rank-sum test, Wilcoxon signed rank-sum test, Fisher’s exact test, and Spearman’s rank-order correlation, with no correction for multiple tests (Sigma Plot 11.0, Jandel Scientific; San Jose, CA). A two-tailed *p* value < 0.05 was considered statistically significant.

## Results

We enrolled fifteen patients with COVID-19 on invasive mechanical ventilation. Their main characteristics at ICU admission are reported in Table 1 and Additional file 1: Table S2. Two (13%) were active smokers, and none (0%) had a history of chronic lung disease.

**Table 1:** Characteristics of the study population at ICU admission

Variable	Study population
*N*	15
Males (*n* [%])	11 (73)
Age (years)	69 (65–74)
Body mass index (BMI) (kg/m^2^)	29 (25–31)
Tidal volume (ml)	400 (400–435)
Tidal volume (ml/kg of PBW)	6.4 (6.0–7.1)
Respiratory rate (bpm)	18 (16–22)
PEEP (cmH_2_O)	12 (10–15)
FiO_2_ (%)	70 (60–88)
Minute ventilation (L/min)	7.6 (6.4–9.0)
Plateau airway pressure (cmH_2_O)	23 (18–25)
Driving airway pressure (cmH_2_O)	9 (7–12)
Compliance (ml/cmH_2_O)	49 (35–58)
Arterial pH	7.37 (7.31–7.40)
PaCO_2_ (mmHg)	55 (43–61)
PaO_2_ (mmHg)	83 (71–108)
PaO_2_:FiO_2_ (mmHg)	123 (91–139)
ICU length of stay (days)	20 (11–42)
Mortality in ICU (*n* [%])	6 (40)

All data refer to the time of admission to our Intensive Care Unit (ICU), except for ICU length of stay and mortality in ICU. BMI—body mass index; PBW—predicted body weight; PEEP—positive end-expiratory pressure; FiO_2_—inspiratory fraction of oxygen; PaCO_2_—arterial tension of carbon dioxide; PaO_2_, arterial tension of oxygen. The driving airway pressure was the difference between the plateau airway pressure and total PEEP measured with a 5-s end-inspiratory and end-expiratory pause. The compliance was the ratio of the tidal volume to the driving airway pressure. Data are reported as median (Q1–Q3) or proportion

The study was performed 4 (3–5) days after hospital admission and 2 (1–2) days after endotracheal intubation. By that time, in the supine position and with PEEP of 12 (10–14) cmH_2_O, the ratio of arterial oxygen tension (PaO_2_) to FiO_2_ was 107 (84–173) mmHg. Seven patients were studied with a FiO_2_ increased to 100% during prone positioning. One patient had a PaO_2_:FiO_2_ of 273 mmHg. We decided to prone him in the radiology unit after noting significant ventral hyperinflation on the lung CT obtained in the supine position, hoping to divert ventilation towards the dorsum, as in ARDS unrelated to COVID-19 [3]. Eleven (73%) patients were studied during their first prone positioning, three (20%) during their second, and one (7%) during his third.

### Lung morphological response to prone positioning

#### Global inflation

In the supine position, the total lung volume was 3277 (2390–3533) ml. Four-hundred-and-seven (238–641) ml or 13 (9–22)% of that total lung volume were in the non-aerated compartment; 729 (563–1181) ml or 25 (19–32)% in the poorly-aerated compartment; 1449 (1189–2142) ml or 50 (42–66)% in the normally-aerated compartment; and 31 (21–376) ml or 1.6 (0.8–10.7)% in the over-aerated compartment. The lung weight was 1434 (1079–1872) g, 619 (221–834) g higher than the estimated premorbid one (Additional file 1: Fig. S1). The lung gas volume was 1541 (1242–2081) ml.

**Fig. 1:**
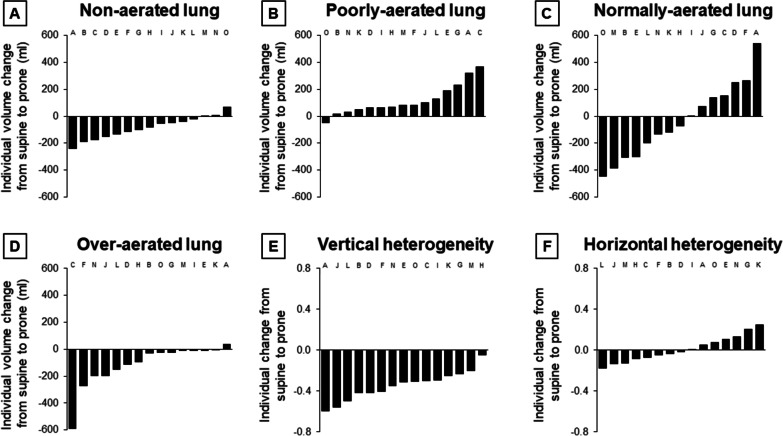
Individual morphological response to prone positioning. Fifteen mechanically-ventilated patients with COVID-19 underwent a lung computed tomography in the supine and prone position. **A**–**D** we describe the individual changes in the total (tissue and gas) volume of the non-aerated (density above − 100 HU), poorly-aerated (from − 100 to − 500 HU), normally-aerated (from − 500 to − 900 HU), and over-aerated (below − 900 HU) compartments with prone positioning, in descending order. **E**, **F** each CT slice was divided into ten equal vertical levels, from the sternum (vertical level 1) to the vertebra (vertical level 10), and in ten equal horizontal levels, from the apex (horizontal level 1) to the base (horizontal level 10) of the lung. Herein we describe the individual change in the degree of heterogeneous aeration along the vertical and horizontal axis, expressed with the standardized median absolute deviation of regional gas-to-tissue ratios, and presented in descending order. Negative values indicate that the volume of a given compartment or the degree of heterogeneity decreased with prone positioning. Each bar refers to one patient. The same letter in the six panels refers to the same patient. Patient N had a baseline PaO_2_:FiO_2_ of 273 mmHg; the decision to prone him was based on the detection of large ventral lung hyperinflation at the CT taken in the supine position (please refer to the main text for other details)

**Fig. 2:**
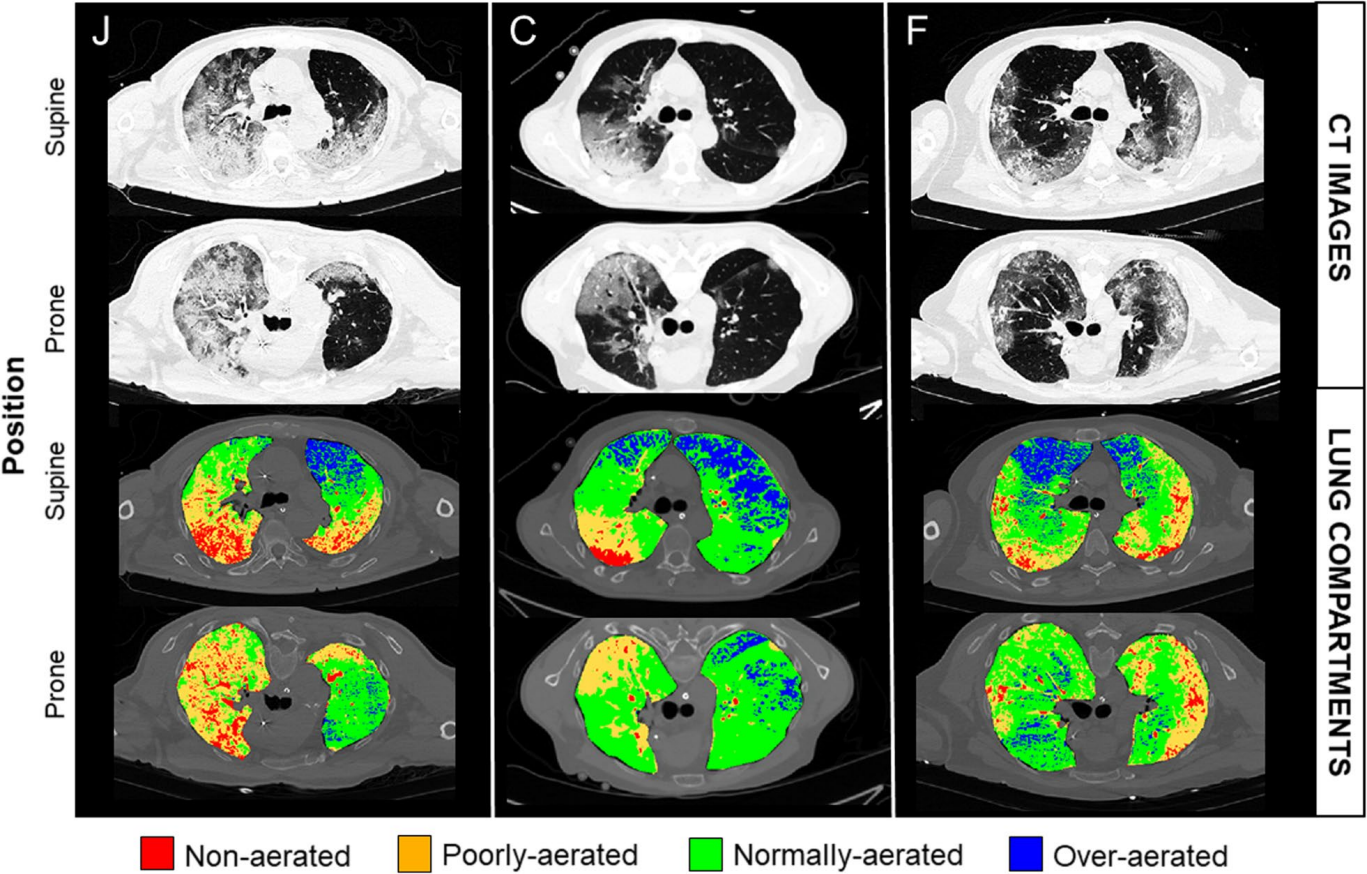
Colour-coded analysis of lung computed tomography (CT) data. Representative CT images taken at the level of carina from three patients with COVID-19 in the supine and prone position, with a very large decrease in the volume of the over-aerated compartment in response to prone positioning. Upper panels: original lung CT images, with aeration shown on a continuous grayscale. Lower panels: using an automated encoding system, we attributed a specific colour to the non-aerated, poorly-aerated, normally-aerated, and over-aerated compartments. The three patients are identified with the same letters as in other figures. With prone positioning, the volume of the over-aerated lung decreased from 318 to 121 ml in patient J; from 738 to 148 ml in patient C; from 503 to 230 ml in patient F

With prone positioning, lung inflation changed as described in Fig. 1, Additional file 1: Tables S3 and S4. The volume of the non-aerated compartment decreased in twelve (80%) patients, and the volume of the over-aerated compartment in fourteen (93%). The total lung volume and weight did not significantly change, while the gas volume decreased by 197 (8–290) ml. The volume of the non-aerated compartment decreased by 82 (26–147) ml or 15 (5–53)%; of the poorly-aerated compartment increased by 82 (53–174) ml (or 9 (6–25)%; of the normally-aerated compartment did not significantly change; of the over-aerated compartment decreased by 28 (11–186) ml or 68 (46–81)% (Fig. 2). In eight (53%) patients, hyperinflation decreased more than alveolar collapse. The gas-to-tissue ratio of the whole lung decreased in twelve (80%) patients (Fig. 1), and from 1.6 (0.7–2.7) to 1.4 (0.8–1.9) ml/g (*p* = 0.008) in the overall study population.

#### Regional inflation

**Fig. 3:**
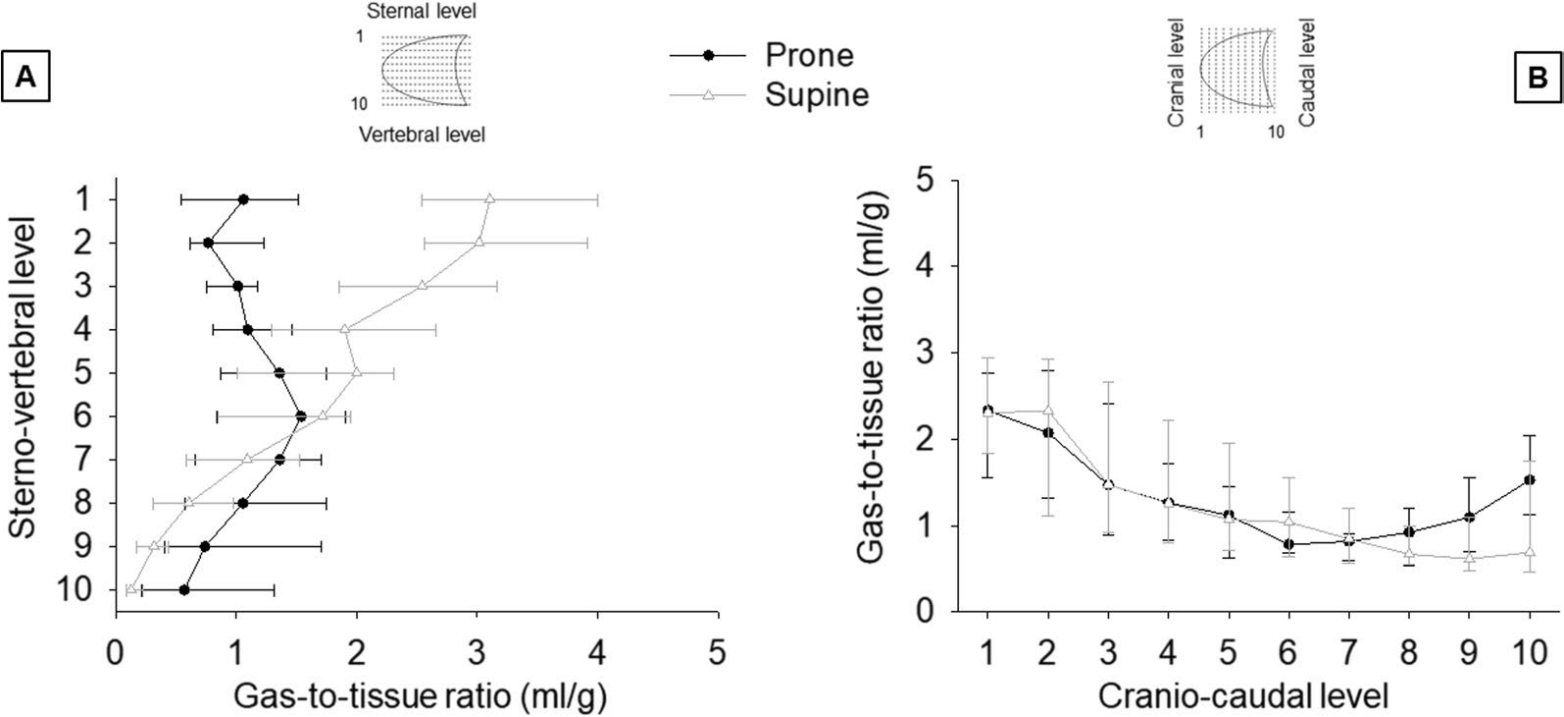
Regional lung gas-to-tissue ratio in the supine and prone position. Fifteen mechanically-ventilated patients with COVID-19 underwent a lung computed tomography (CT) in supine and prone positions. Each CT slice was divided into ten equal vertical levels, from the sternum (vertical level 1) to the vertebra (vertical level 10), and in ten equal horizontal levels, from the apex (horizontal level 1) to the base (horizontal level 10) of the lung. Herein we describe the ratio of the gas volume (ml) to the tissue weight (g) in each of those levels, in the supine and prone positions. Data are reported as median (Q1–Q3). **A** vertical gradient of lung inflation. On average, the gas-to-tissue ratio decreased by 0.34 (0.25–0.49) ml/g per level in the supine position and by 0.03 (− 0.11 to 0.14) ml/g in the prone position (*p* < 0.001). **B** horizontal gradient of lung inflation. On average, the gas-to-tissue ratio decreased by 0.14 (0.04–0.27) ml/g per level in the supine position and by 0.11 (− 0.05 to 0.21) ml/g in the prone position (*p* = 0.003)

In the supine position, the over-aerated compartment tended to be larger ventrally and cranially while the non-aerated compartment dorsally and caudally (Additional file 1: Figs. S2 and S3). The gas-to-tissue ratio decreased from the sternum to the vertebra and from the apex to the base of the lung (Fig. 3). The distribution of inflation was more heterogeneous along the vertical than horizontal axis, with a standardized median absolute deviation of 0.55 (0.50–0.71) and 0.31 (0.16–0.47), respectively (*p* = 0.012). The superimposed pressure partly explained the vertical gradient of aeration: it progressively increased from 0.5 (0.4–0.6) cmH_2_O close to the sternum to 9.1 (7.2–11.3) cmH_2_O close to the vertebra (Additional file 1: Fig. S4).

With prone positioning, the volume of the over-aerated compartment decreased in ventral regions and from the apex to the base of the lung (Additional file 1: Figs. S2 and S3). The volume of the non-aerated compartment decreased in dorsal and caudal regions and increased in ventral ones, although to a minor degree. The gas-to-tissue ratio remained variable throughout the lung (Fig. 3), but the degree of heterogeneity decreased along the vertical axis in all (100%) patients and along the horizontal axis in eight (53%). The standardized median absolute deviation decreased from the sternum to the vertebra (from 0.55 [0.50–0.71] to 0.20 [0.14–0.27], *p* < 0.001); it did not change from the apex to the base of the lung (from 0.31 [0.16–0.47] to 0.29 [0.22–0.46]; *p* = 0.934) (Fig. 3). Changes in the vertical gradient of aeration were associated with those of the superimposed pressure (Additional file 1: Fig. S5).

### Lung functional response to prone positioning

Once in the ICU, fourteen patients were studied in supine and prone positions. One was not because an acute and severe cardiac arrhythmia contraindicated prone positioning. The ventilatory setting remained the same from supine to prone in ten patients. In four others, it was slightly modified: the FiO_2_ was increased (*n* = 1) or decreased (*n* = 2), or the respiratory rate was increased (*n* = 1) during prone positioning (Additional file 1: Table S5).

**Fig. 4:**
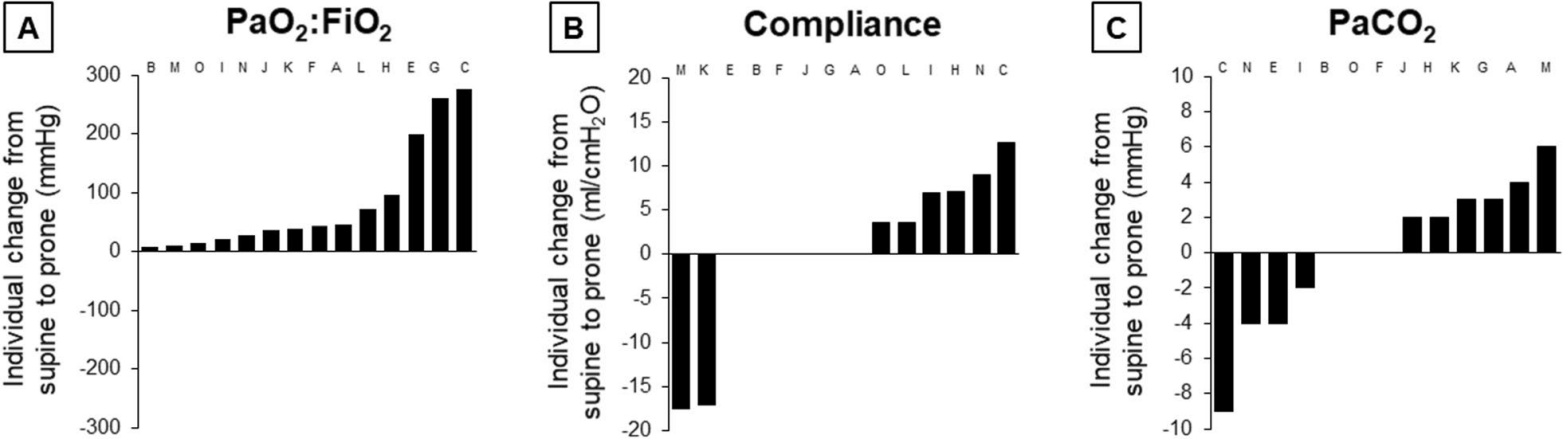
Individual functional response to prone positioning. Fourteen mechanically-ventilated patients with COVID-19 were evaluated in the supine and prone positions. Herein we describe the response to prone positioning in terms of change in arterial oxygenation (expressed as the ratio of the arterial tension to the inspiratory fraction of oxygen [PaO_2_:FiO_2_]) (*n* = 14) (**A**), respiratory system compliance (*n* = 14) (**B**), and carbon dioxide tension (PaCO_2_) for the same minute ventilation (*n* = 13) (**C**), in descending order. Each bar refers to one patient. The same letter in the three panels refers to the same patient. Please note that patient D, present in other figures, did not undergo prone positioning and is absent from this figure. FiO_2_ was decreased in the prone position in patient K and patient F, and increased in patient G. The impact of prone positioning on PaCO_2_ could not be assessed in patient L because his minute ventilation was increased during prone positioning. Finally, patient N had a baseline PaO_2_:FiO_2_ of 273 mmHg; the decision to prone him was based on the detection of large ventral lung hyperinflation at the CT taken in the supine position (please refer to the main text for other details)

With prone positioning, the PaO_2_:FiO_2_ improved in all fourteen (100%) patients and by ≥ 20 mmHg in eleven (79%). Compliance increased in six (43%), remained constant in six (43%), and decreased in two (14%). The arterial carbon dioxide tension (PaCO_2_) increased in six (46%), remained constant in three (23%), and decreased in four (31%) of the thirteen patients with unchanged respiratory rate and minute ventilation (Fig. 4). On average, the PaO_2_:FiO_2_ increased by 41 (21–97) mmHg while compliance and PaCO_2_ did not significantly change (Additional file 1: Tables S4 and S5).

### Association between the morphological and functional response to prone positioning

The change in volume of the non-aerated compartment was associated with neither the severity of the alveolar collapse in the supine position (rho 0.375, *p* = 0.162) nor the concomitant change in PaO_2_:FiO_2_ (rho − 0.415, *p* = 0.134) (Additional file 1: Fig. S6), compliance (rho 0.062, *p* = 0.820) or PaCO_2_ (rho 0.094, *p* = 0.751).

The change in volume of the over-aerated compartment was strongly associated with the degree of hyperinflation in the supine position (rho − 0.961, *p* < 0.001) (Fig. 2 and Additional file 1: Fig. S7). It also tended to be associated with the concomitant change in compliance (rho − 0.541, *p* = 0.045) and PaCO_2_ (rho 0.491, *p* = 0.085), but not PaO_2_:FiO_2_ (rho − 0.130, *p* = 0.648) (Additional file 1: Fig. S8).

The change in the heterogeneity of lung aeration along the vertical axis tended to be associated with its value in the supine position (rho − 0.468, *p* = 0.076) but not with the concomitant change in PaO_2_:FiO_2_ (rho 0.108, *p* = 0.704), compliance (rho 0.021, *p* = 0.940) or PaCO_2_ (rho 0.191, *p* = 0.516).

## Discussion

The lung response to prone positioning was variable in patients with early ARDS due to COVID-19. In general, the volume of the non-aerated and over-aerated tissue decreased, and the distribution of aeration became more homogeneous; arterial oxygenation improved, but compliance and PaCO_2_ did not.

In ARDS unrelated to COVID-19, prone positioning decreases alveolar collapse and hyperinflation and homogenizes the distribution of end-expiratory aeration and tidal inflation [3–5]. As a result, mechanical ventilation generates less alveolar deformation and tension and less pulmonary damage [20–25]. This is the strongest rationale for prone positioning in ARDS: making mechanical ventilation safer [6, 7, 20–25]. Increasing arterial oxygenation is probably less important [26–28] except for the unusual case of life-threatening hypoxemia.

In early ARDS due to COVID-19, the lung morphological response to prone positioning resembled that in other ARDS. Alveolar collapse and hyperinflation decreased, and the distribution of aeration became more homogeneous. In the supine position, and from the sternum to the vertebra, the regional gas-to-tissue ratio ranged from 3.1 (2.5–4.0) to 0.1 (0.1–0.2) ml/g; in the prone position, and from the vertebra to the sternum, from 1.5 (0.8–1.9) to 0.6 (0.2–1.3) ml/g (Fig. 3). Therefore, the peak value and dispersion of inflation along the vertical axis were smaller in prone than supine position. Changes in the horizontal distribution of aeration were usually minor. Based on these findings, prone positioning may protect patients with COVID-19 from secondary lung damage [29], as it does in other ARDS.

Several factors probably contributed to redistributing lung aeration with prone positioning. As shown in Additional file 1: Fig. S5, one of these factors was the superimposed pressure [19]: the gas-to-tissue ratio increased, did not change, or decreased where the superimposed pressure decreased, remained constant, or increased, respectively [3]. Other possible factors include (1) the shape of the lung and the chest wall [30, 31]; (2) the compression of the lung by the heart and the abdomen [32, 33]; (3) the compliance of the non-dependent and dependent rib cage [34]; and (4) the vertical distribution of the lung mass [6].

With prone positioning, arterial oxygenation almost always increased while the volume of the non-aerated lung decreased. Nonetheless, these two responses were unrelated in magnitude. With COVID-19, the distribution of the pulmonary blood flow can be very heterogeneous [35, 36]. For a given lung recruitment, oxygenation will increase more or less if the newly aerated alveoli are hyper or hypo-perfused. This can be why, in our study population, the reversal of alveolar collapse was not always associated with a proportional increase in arterial oxygenation. None of the patients had documented pulmonary thrombosis. However, as only two of them underwent a lung CT with contrast, the others may still have had some unrecognized pulmonary perfusion defects.

Changes in compliance and PaCO_2_ were partly associated with those in hyperinflation. With a larger decrease in the volume of the over-aerated lung, respiratory system compliance increased. As the chest wall compliance reasonably decreased [34], lung compliance probably increased even more. At the same time, PaCO_2_ tended to decrease. These data suggest that hyperinflation at lung CT was associated with overdistention and that prone positioning decreased both. However, several poorly predictable factors can confound the interpretation of an individual response to prone positioning. For example, the change in respiratory system compliance can also depend on the behaviour of the chest wall, and the change in dead space and PaCO_2_ on the distribution of the pulmonary blood flow [6].

Hyperinflation is common in patients with COVID-19, even those ventilated with low tidal volume and airway pressure [16, 37, 38]. In the seven patients with a larger (than the median) volume of the over-aerated compartment, tidal volume was 6.1 (5.7–6.5) ml/kg of predicted body weight, and plateau airway pressure 23 (21–23) cmH_2_O (Additional file 1: Table S6). Hyperinflation is a well-known risk factor for secondary lung damage [39, 40]. In our previous study [16], increasing PEEP from 5 to 15 cmH_2_O in the supine position decreased the volume of the non-aerated lung by 168 (110–202) ml but increased the volume of the over-aerated lung by 121 (63–270) ml. Hyperinflation increased with a higher PEEP in all (100%) patients. Herein, prone positioning decreased the volume of the non-aerated lung by 82 (26–147) ml and the volume of the over-aerated compartment by 28 (11–186) ml. Hyperinflation *decreased* in all patients but one (93%), especially in those with a larger over-aerated compartment when supine. Therefore, prone positioning may recruit the lung with less hyperinflation than a higher PEEP.

So far, the morphological and functional response to prone positioning in COVID-19 has been investigated only partially [41–43]. Herein we show that with prone positioning: (1) aeration is globally more evenly distributed so that harms from mechanical ventilation should be reduced; (2) a “beneficial” morphological response cannot be predicted from changes in gas exchange and respiratory system mechanics; (3) the decrease in hyperinflation (herein measured as the *volume* of the over-aerated lung) is frequently larger than recruitment. This can be particularly important in patients with COVID-19, who are at an increased risk of ventilator-induced lung damage [38].

Some of the limitations of this study deserve a comment. First, we could not enrol all consecutive eligible patients during the first pandemic wave, which may have been a source of bias (see Additional file 1: Table S1). Second, data were analysed with no correction for multiple tests, so our results should be considered preliminary. Third, the lung CTs were obtained at end-expiration, and we did not study the distribution of tidal volume in the supine and prone positions. Fourth, our study design differed in many aspects from common clinical practise. Lung response was assessed soon after prone positioning. However, patients are usually kept prone for several hours, during which their response can evolve [2]. A recruitment manoeuvre was always performed before and after prone positioning, which may not be part of routine care [2]. PEEP was set at the discretion of the attending physician; if set differently, lung morphology and function would have probably differed [16]. All of these issues limit the generalizability of our findings. Fifth, the effects of prone positioning may not be the same in patients with late COVID-19 [42]. Finally, we did not study the impact of prone positioning on patient-centred outcomes, such as survival or duration of mechanical ventilation.

## Conclusions

In this preliminary physiological study on fifteen mechanically-ventilated patients with early COVID-19, prone positioning variably decreased the amount of alveolar collapse and hyperinflation and improved the distribution of aeration and arterial oxygenation. A similar response has been observed in other ARDS, where prone positioning improves outcome. Therefore, our data provide a pathophysiological rationale to support prone positioning in COVID-19.

## Supplementary Information

**Additional file 1**. Online data supplement.

## Data Availability

The datasets used and analysed during the current study are available from the corresponding author on reasonable request.

## Abbreviations

ARDS: Acute respiratory distress syndrome
COPD: Chronic obstructive pulmonary disease
COVID-19: Coronavirus disease 2019
CT: Computed tomography
FiO_2_: Fraction of inspired oxygen
ICU: Intensive care unit
PaCO_2_: Arterial carbon dioxide tension
PaO_2_: Arterial oxygen tension
PEEP: Positive end-expiratory pressure
Q1: First quartile
Q3: Third quartile
