# Supplementary material for: Lung response to prone positioning in mechanically-ventilated patients with COVID-19
Source: Crit Care. 2022 May 7;26:127. doi: 10.1186/s13054-022-03996-0 (PMC9076814; doi:10.1186/s13054-022-03996-0)
Supplement: Supplementary file 1 — Additional file 1. Online data supplement. [file 13054_2022_3996_MOESM1_ESM.docx]

Online data supplement

**Lung response to prone positioning in mechanically ventilated patients with COVID-19**

Alessandro Protti, Alessandro Santini, Francesca Pennati, Chiara Chiurazzi, Michele Ferrari, Giacomo E. Iapichino, Luca Carenzo, Francesca Dalla Corte, Ezio Lanza, Nicolò Martinetti, Andrea Aliverti, Maurizio Cecconi

**Table S1.** Comparison between patients treated with prone positioning and included or excluded from our study.

| **Variable** | **Included** | **Excluded** | **P-value** |
| --- | --- | --- | --- |
| N | 15 | 46 |  |
| *General characteristics* | | | |
| Males (n [%]) | 11 (73) | 33 (72) | >0.999 |
| Age (years) | 69 (65-74) | 64 (54-70) | 0.029 |
| Body mass index (BMI) (kg/m^2^) | 29 (25-31) | 28 (25-33) | 0.993 |
| *Ventilatory setting* | | | |
| Tidal volume (ml) | 400 (400-435) | 420 (380-473)* | 0.643 |
| Tidal volume (ml/kg of PBW) | 6.4 (6.0-7.1) | 6.4 (5.9-7.0)* | 0.973 |
| Respiratory rate (bpm) | 18 (16-22) | 22 (20-27) | <0.001 |
| PEEP (cmH_2_O) | 12 (10-15) | 12 (12-14) | 0.550 |
| FiO_2_ (%) | 70 (60-88) | 70 (60-90) | 0.563 |
| Minute ventilation (L/min) | 7.6 (6.4-9.0) | 9.7 (8.4-11.3)* | 0.001 |
| *Respiratory system mechanics* | | | |
| Plateau airway pressure (cmH_2_O) | 23 (18-25) | 24 (22-27)° | 0.092 |
| Driving airway pressure (cmH_2_O) | 9 (7-12) | 11 (8-13)° | 0.065 |
| Compliance (ml/cmH_2_O) | 49 (35-58) | 37 (32-45)° | 0.096 |
| *Gas exchange* | | | |
| Arterial pH | 7.37 (7.31-7.40) | 7.38 (7.30-7.46) | 0.356 |
| PaCO_2_ (mmHg) | 55 (43-61) | 47 (40-54) | 0.109 |
| PaO_2_ (mmHg) | 83 (71-108) | 78 (65-92) | 0.200 |
| PaO_2_:FiO_2_ (mmHg) | 123 (91-139) | 113 (93-147) | 0.627 |
| *Outcomes* | | | |
| ICU length of stay (days) | 20 (11-42) | 18 (10-31) | 0.718 |
| Mortality in ICU (n [%]) | 6 (40) | 21 (46) | 0.771 |

All data refer to the time of admission to our Intensive Care Unit (ICU), except for outcomes. BMI: body mass index. PBW: predicted body weight. PEEP: positive end-expiratory pressure. FiO_2_: inspiratory fraction of oxygen. PaCO_2_: arterial tension of carbon dioxide. PaO_2_: arterial tension of oxygen. The driving airway pressure was the difference between the plateau airway pressure and total PEEP measured with a 5-second end-inspiratory and end-expiratory pause. The compliance was the ratio of the tidal volume to the driving airway pressure. Data are reported as median (Q1-Q3). P-value refers to the Wilcoxon signed rank-sum test or the Fisher’s exact test. * One missing value. ° Two missing values.

**Table S2.** Additional data on the study population.

| **Variable** | **Included** |
| --- | --- |
| N | 15 |
| *General characteristics* | |
| - Current smoker (n [%]) | 2 (13) |
| History of COPD (n [%]) | 0 (0) |
| Time from symptom onset to hospital admission (days) | 7 (6-10) |
| Time from hospital admission to intubation (days) | 2 (1-4) |
| Time from symptom onset to intubation (days) | 10 (8-16) |
| Time from intubation to CT (days) | 2 (1-2) |
| Time from symptom onset to CT (days) | 11 (10-17) |
| *Hemodynamics* | |
| Heart rate (beats/minute) | 80 (79-88) |
| Mean arterial pressure (mmHg) | 80 (70-89) |
| Arterial lactate (mmol/L) | 1.1 (0.9-1.4) |
| With catecholamine(s) (n [%]) | 7 (47) |
| *Blood biochemistry* | |
| White blood cells (*10^9^/L) | 12.1 (9.0-13.0) |
| C-reactive protein (mg/dl) | 12.1 (5.1-28.7) |
| Procalcitonin (ng/ml) | 0.5 (0.1-1.2) |
| D-dimer (ng/ml) | 1335 (817-4041) |
| Bilirubin (mg/dl) | 0.8 (0.4-1.3) |
| Creatinine (mg/dl) | 0.9 (0.6-1.0) |
| Platelets (*10^3^/L) | 240 (205-363) |

These data complement those presented in Table 1 in the main manuscript. They all refer to those same fifteen mechanically-ventilated patients with COVID-19 at the time of admission to our Intensive Care Unit. COPD: chronic obstructive pulmonary disease. CT: (lung) computed tomography. Data are reported as median (Q1-Q3) or proportion.

**Figure S1.** Relationship between excess lung weight and circulating C-reactive protein.


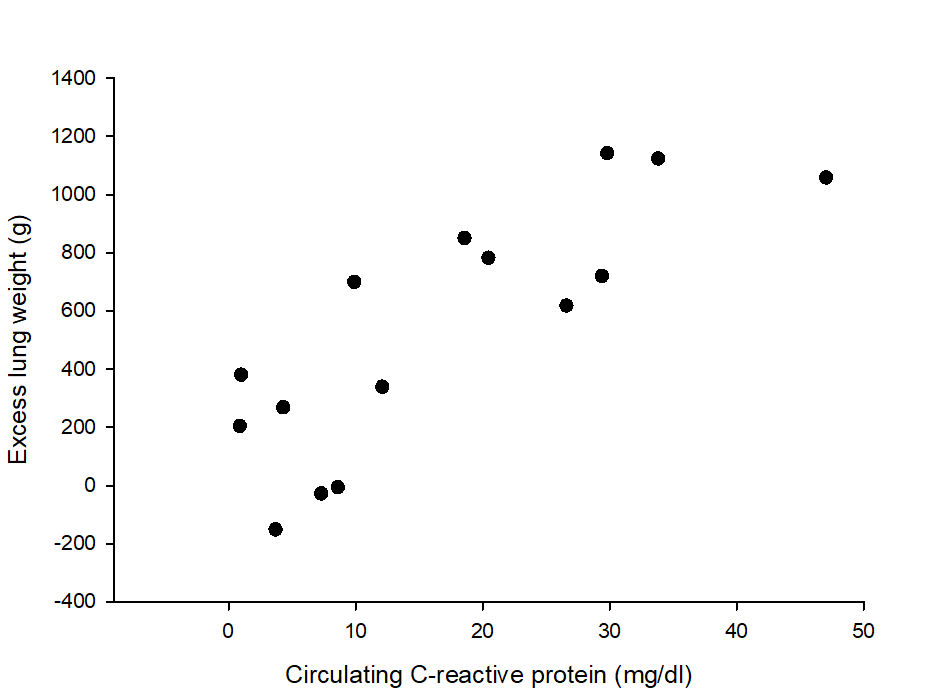


Fifteen patients with COVID-19 requiring mechanical ventilation underwent a lung computed tomography (CT) within 2 (1-2) days after endotracheal intubation. Herein we show the association between the excess lung weight, that reflects the degree of pulmonary edema, and the circulating C-reactive protein, a marker of systemic inflammation (rho 0.829; p<0.001). The excess lung weight was computed as the difference between the actual lung weight, measured with the lung CT (as reported in the main manuscript) and the premorbid lung weight, estimated on the basis of sex and height (E1).

**Table S3.** Lung morphological response to prone positioning.

| **Variable** | | **Supine** | **Prone** | **P-value** |
| --- | --- | --- | --- | --- |
| N | | 15 | 15 |  |
| *Lung tissue and gas distribution* | | | | |
| Total lung | Total (ml) | 3277 (2390-3533) | 3077 (2266-3624) | 0.073 |
|  | Tissue (g) | 1434 (1079-1872) | 1455 (1078-1839) | 0.524 |
|  | Gas (ml) | 1541 (1242-2081) | 1460 (1123-1935) | 0.041 |
| Non-aerated | Total (ml) | 407 (238-641) | 336 (113-547) | 0.003 |
|  | Tissue (g) | 412 (232-636) | 332 (109-534) | NT |
|  | Gas (ml) | 3.6 (0.0-6.9) | 1.3 (0.1-3.8) | NT |
| Poorly-aerated | Total (ml) | 729 (563-1181) | 885 (672-1293) | <0.001 |
|  | Tissue (g) | 515 (382-817) | 611 (449-887) | NT |
|  | Gas (ml) | 230 (165-365) | 274 (213-431) | NT |
| Normally-aerated | Total (ml) | 1449 (1189-2142) | 1438 (1209-2130) | 0.599 |
|  | Tissue (g) | 385 (345-532) | 450 (328-609) | NT |
|  | Gas (ml) | 1079 (843-1537) | 1059 (858-1491) | NT |
| Over-aerated | Total (ml) | 31 (21-376) | 41 (4-141) | 0.002 |
|  | Tissue (g) | 2.7 (1.5-17.0) | 3.4 (0.2-7.9) | NT |
|  | Gas (ml) | 29 (19-360) | 37 (3-129) | NT |
| Non-aerated | Total (%) | 13 (9-22) | 12 (4-15) | 0.041 |
|  | Tissue (%) | 29 (23-36) | 12 (4-15) | NT |
|  | Gas (%) | 0.2 (0.0-0.4) | 0.1 (0.0-0.2) | NT |
| Poorly-aerated | Total (%) | 25 (19-32) | 33 (24-35) | <0.001 |
|  | Tissue (%) | 36 (32-46) | 43 (37-51) | NT |
|  | Gas (%) | 16 (10-22) | 20 (14-26) | NT |
| Normally-aerated | Total (%) | 50 (42-66) | 57 (41-65) | 0.454 |
|  | Tissue (%) | 31 (21-41) | 33 (22-41) | NT |
|  | Gas (%) | 71 (62-83) | 73 (67-82) | NT |
| Over-aerated | Total (%) | 1.6 (0.8-10.7) | 1.2 (0.2-3.6) | 0.001 |
|  | Tissue (%) | 0.4 (0.1-1.2) | 0.3 (0.0-0.6) | NT |
|  | Gas (%) | 3.4 (1.7-19.9) | 2.1 (0.4-6.7) | NT |

Fifteen mechanically-ventilated patients with COVID-19 underwent a lung computed tomography in the supine and prone position. Herein we describe the average changes in the total (tissue and gas) volume, tissue weight, and gas volume of the whole lung and its non-aerated, poorly-aerated, normally-aerated, and over-aerated compartments with prone positioning. Data are reported as median (Q1-Q3) or proportion. P-value refers to the Wilcoxon signed rank-sum test. To limit the number of tests, we only compared data of primary interest: total (tissue and gas) volume, tissue weight and gas volume of the whole lung, and total volume of its 4 compartments. NT: not tested. Please note that the volume of the over-aerated compartment *decreased* with prone positioning (by 28 [11-186] ml on average), as suggested by a much smaller Q1-Q3, and as shown in Figure 1 in the main manuscript. At first sight this may not be clear, as the median volume of the over-aerated compartment looks higher in prone than supine position.

**Table S4.** Individual change in lung morphology and function from supine to prone.

| ID | Non-aerated lung (ml) | Poorly-aerated lung (ml) | Normally-aerated lung (ml) | Over-aerated lung (ml) | Vertical heterogeneity | Horizontal heterogeneity | PaO_2_:FiO_2_ (mmHg) | Compliance (ml/cmH_2_O) | PaCO_2_ (mmHg) |
| --- | --- | --- | --- | --- | --- | --- | --- | --- | --- |
| A | -242 | 320 | 539 | 35 | -0.59 | 0.05 | 45 | 0 | 4 |
| B | -188 | 17 | -305 | -28 | -0.42 | -0.03 | 9 | 0 | 0 |
| C | -175 | 369 | 153 | -590 | -0.30 | -0.07 | 276 | 13 | -9 |
| D | -151 | 61 | 252 | -115 | -0.41 | -0.01 | NA | NA | NA |
| E | -135 | 189 | -300 | -9 | -0.31 | 0.11 | 200 | 0 | -4 |
| F | -115 | 84 | 263 | -273 | -0.40 | -0.05 | 42 | 0 | 0 |
| G | -98 | 233 | 137 | -23 | -0.23 | 0.21 | 262 | 0 | 3 |
| H | -82 | 68 | -72 | -96 | -0.05 | -0.08 | 97 | 7 | 2 |
| I | -53 | 64 | 2 | -10 | -0.29 | 0.00 | 21 | 7 | -2 |
| J | -50 | 99 | 71 | -197 | -0.56 | -0.13 | 36 | 0 | 2 |
| K | -41 | 50 | -118 | -6 | -0.25 | 0.25 | 39 | -17 | 3 |
| L | -21 | 128 | -201 | -152 | -0.50 | -0.17 | 72 | 4 | NA |
| M | 1 | 82 | -385 | -11 | -0.20 | -0.13 | 10 | -18 | 6 |
| N | 8 | 30 | -134 | -197 | -0.35 | 0.13 | 27 | 9 | -4 |
| O | 69 | -48 | -447 | -27 | -0.30 | 0.08 | 14 | 4 | 0 |

The morphological response to prone positioning was studied in fifteen mechanically-ventilated patients with COVID-19 as the change in lung aeration from supine to prone. The functional response was assessed in fourteen of them as the change in gas exchange and respiratory system compliance. Herein we describe the data recorded from each patient. NA: not available that is the functional response to prone positioning could not be assessed for reasons detailed in the main manuscript. Patient N had a baseline PaO_2_:FiO_2_ of 273 mmHg; the decision to prone him was based on the detection of large ventral lung hyperinflation at the CT taken in the supine position (please refer to the main text for other details).

**Figure S2.** Regional lung aeration along the sterno-vertebral axis in the supine and prone position.


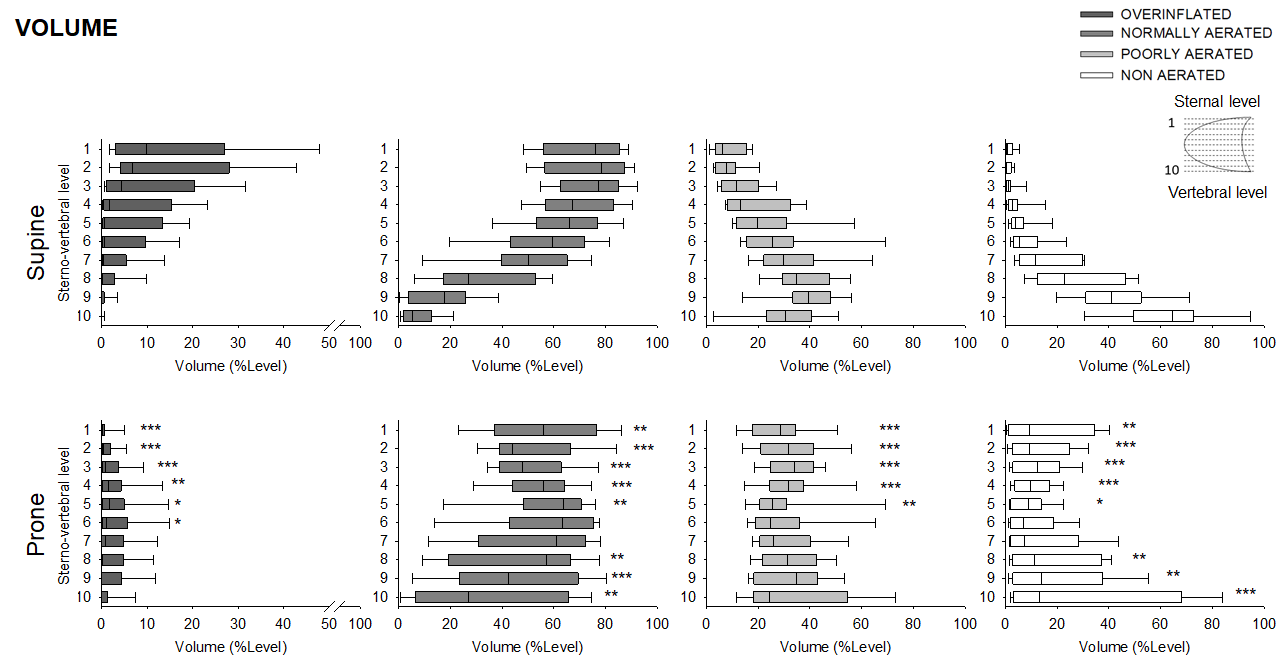


Fifteen mechanically-ventilated patients with COVID-19 underwent a lung computed tomography (CT) in the supine and prone position. Each CT slice was divided in 10 equal vertical levels, from the sternum (level 1) to the vertebra (level 10). Herein we describe the total (tissue and gas) volume of the non-aerated, poorly-aerated, normally-aerated, and over-aerated lung compartments in those 10 levels, in the supine and prone position. Data are reported as box plots. They were compared within subjects with the Wilcoxon signed rank-sum test, with no adjustment for multiple comparisons. * p<0.05, ** p<0.01, *** p<0.001 for the same level in prone compared to supine.

**Figure S3.** Regional lung aeration along the cranio-caudal axis in the supine and prone position.


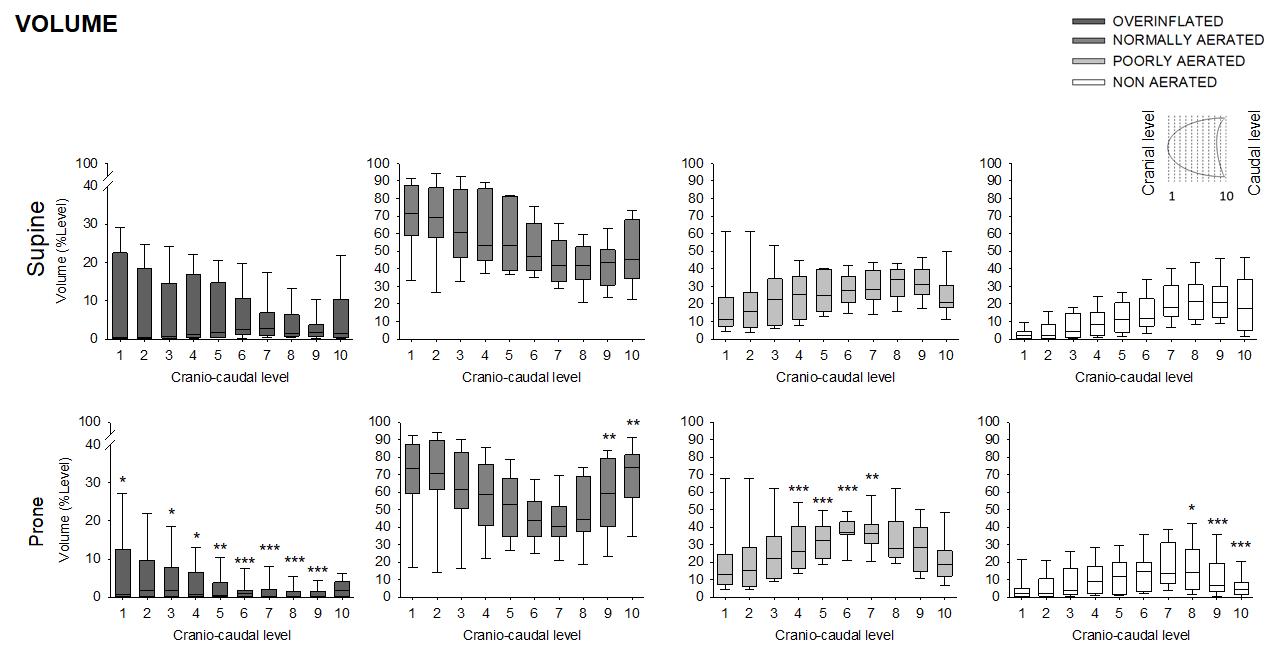


Fifteen mechanically-ventilated patients with COVID-19 underwent a lung computed tomography (CT) in the supine and prone position. Each CT slice was divided in 10 equal horizontal levels, from the apex (level 1) to the base (level 10) of the lung. Herein we describe the total (tissue and gas) volume of the non-aerated, poorly-aerated, normally-aerated, and over-aerated lung compartments in those 10 levels, in the supine and prone position. Data are reported as box plots. They were compared within subjects with the Wilcoxon signed rank sum test, with no adjustment for multiple comparisons. * p<0.05, ** p<0.01, *** p<0.001 for the same level in prone compared to supine.

**Figure S4.** Distribution of the superimposed pressure along the vertical axis of the lung.


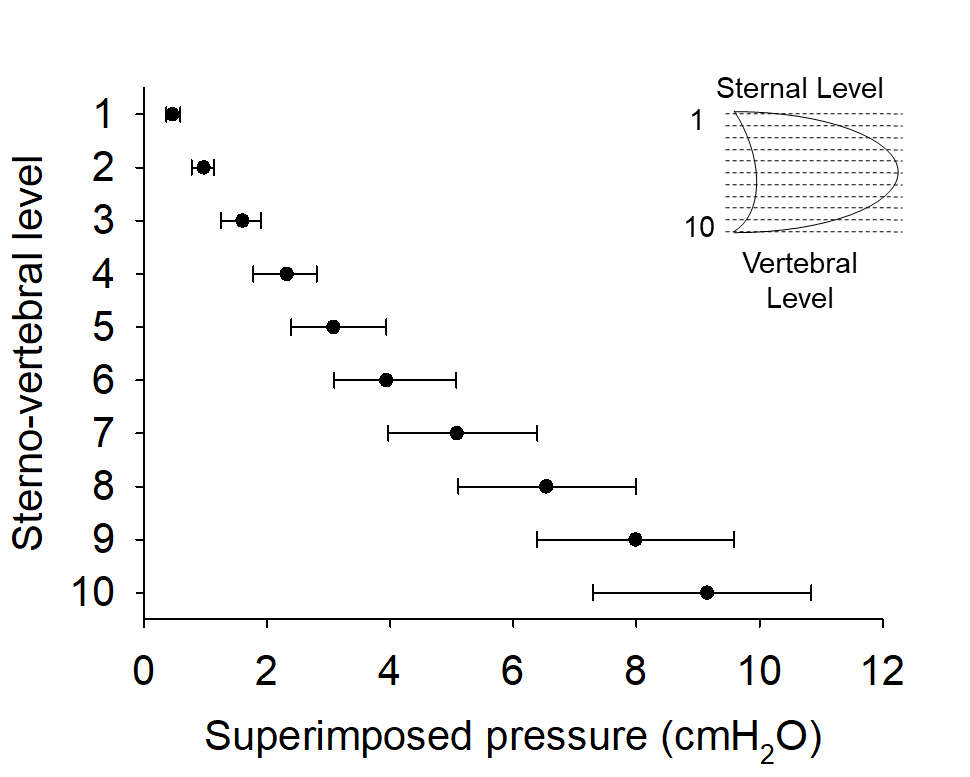


Fifteen mechanically-ventilated patients with COVID-19 underwent a lung computed tomography (CT) in the supine and prone position. Each CT slice was divided in 10 equal vertical levels, from the sternum (level 1) to the vertebra (level 10). Herein we show the distribution of the superimposed pressure along this vertical axis. The superimposed pressure can be defined as the hydrostatic pressure that each lung level exerts on those below in virtue of its weight (typically augmented in ARDS, due to edema) and vertical height (E2). According to the so-called “sponge model”, lung aeration decreases from the sternum to the vertebra because the superimposed pressure progressively increases along the vertical axis of the lung.

**Figure S5.** Change in the superimposed pressure and regional lung inflation in response to prone positioning.


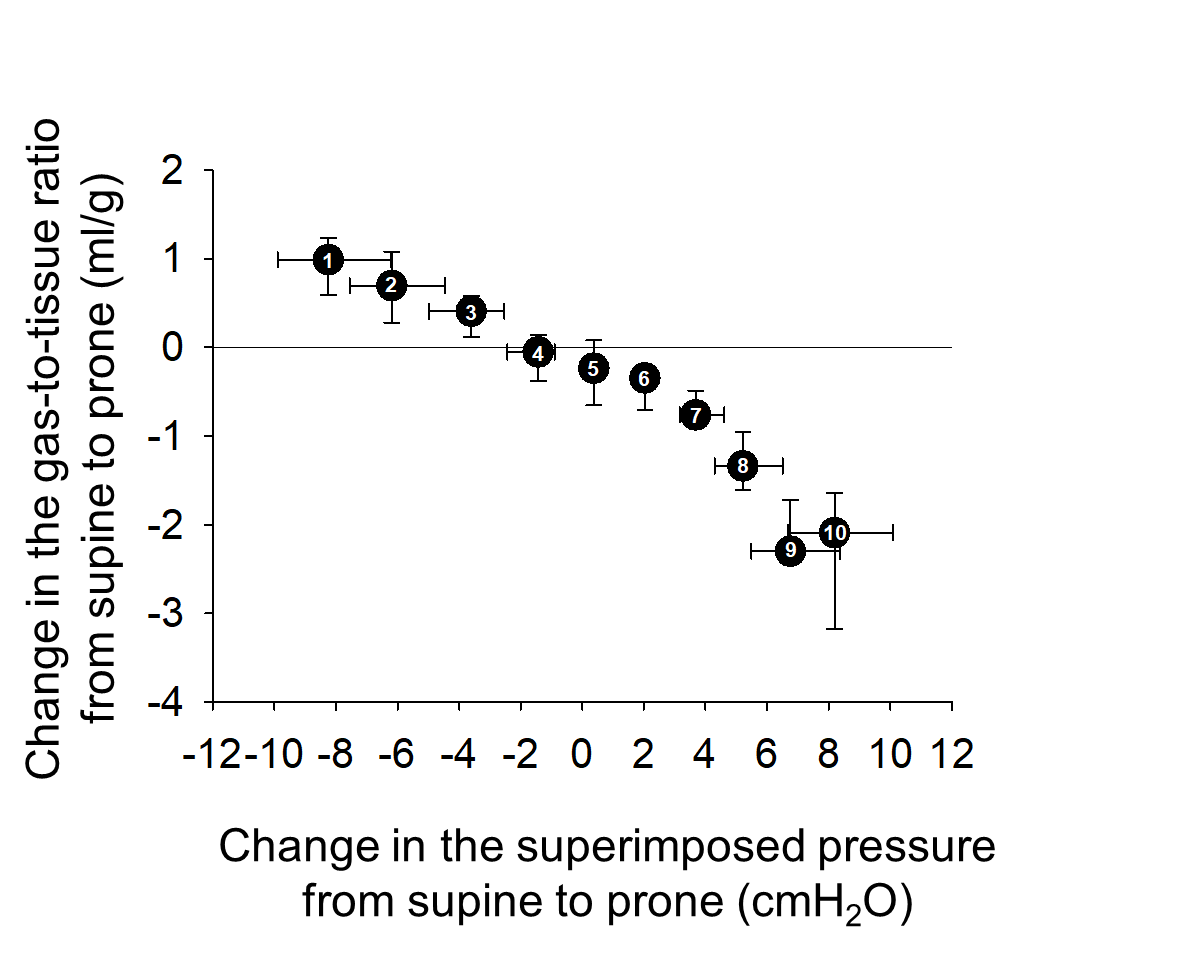


Fifteen mechanically-ventilated patients with COVID-19 underwent a lung computed tomography (CT) in the supine and prone position. Each CT slice was divided in 10 equal vertical levels, from the sternum (level 1) to the vertebra (level 10). Herein we show the association between the change in the superimposed pressure and aeration (expressed as the gas-to-tissue ratio) at each lung level in response to prone positioning (rho -0.988, p<0.001). Numbers on black dots refer to the lung level.

**Table S5.** Lung functional response to prone positioning.

| **Variable** | **Supine** | **Prone** | **P-value** |
| --- | --- | --- | --- |
| N | 14 | 14 |  |
| *Ventilatory setting* | | | |
| Tidal volume (ml) | 400 (370-420) | 400 (370-420) | >0.999 |
| Tidal volume (ml/kg of PBW) | 6.1 (6.0-6.9) | 6.1 (6.0-6.9) | >0.999 |
| Respiratory rate (bpm) | 19 (16-20) | 20 (18-20) | >0.999 |
| PEEP (cmH_2_O) | 12 (10-12) | 12 (10-12) | >0.999 |
| FiO_2_ (%) | 1.0 (0.8-1.0) | 0.9 (0.7-1.0) | >0.999 |
| Minute ventilation (L/min) | 7.6 (6.4-8.8) | 7.8 (6.5-8.8) | >0.999 |
| *Respiratory system mechanics* | | | |
| Plateau airway pressure (cmH_2_O) | 21 (20-23) | 22 (20-23) | 0.469 |
| Driving airway pressure (cmH_2_O) | 8 (7-11) | 9 (8-10) | 0.461 |
| Compliance (ml/cmH_2_O) | 45 (36-57) | 44 (40-56) | 0.742 |
| *Gas exchange* | | | |
| Arterial pH | 7.35 (7.33-7.38) | 7.35 (7.32-7.36) | 0.787 |
| PaCO_2_ (mmHg) | 56 (53-59) | 57 (51-59) | 0.765 |
| PaO_2_ (mmHg) | 84 (63-171) | 148 (77-300) | <0.001 |
| PaO_2_:FiO_2_ (mmHg) | 100 (83-171) | 156 (117-300) | <0.001 |
| SaO_2_ (%) |  |  |  |
| *Hemodynamics* | | | |
| Heart rate (beats/minute)* | 76 (67-84) | 75 (65-83) | 0.244 |
| Mean arterial pressure (mmHg)* | 76 (74-91) | 80 (70-85) | 0.380 |
| Arterial lactate (mmol/L)* | 1.1 (0.9-1.3) | 1.1 (1.0-1.3) | 0.813 |
| ScvO_2_ (%) | 78 (70-85) | 82 (75-87) | 0.042 |

Fourteen mechanically-ventilated patients with COVID-19 were evaluated in the supine and prone position. PBW: predicted body weight. PEEP: positive end-expiratory pressure. FiO_2_: inspiratory fraction of oxygen. PaCO_2_: arterial tension of carbon dioxide. PaO_2_: arterial tension of oxygen. SaO_2_: arterial hemoglobin oxygen saturation. ScvO_2_: central venous hemoglobin oxygen saturation. Data are reported as median (Q1-Q3). P-value refers to the Wilcoxon signed rank-sum test. * One missing value.

**Figure S6.** Alveolar recruitment and change in arterial oxygenation in response to prone positioning.


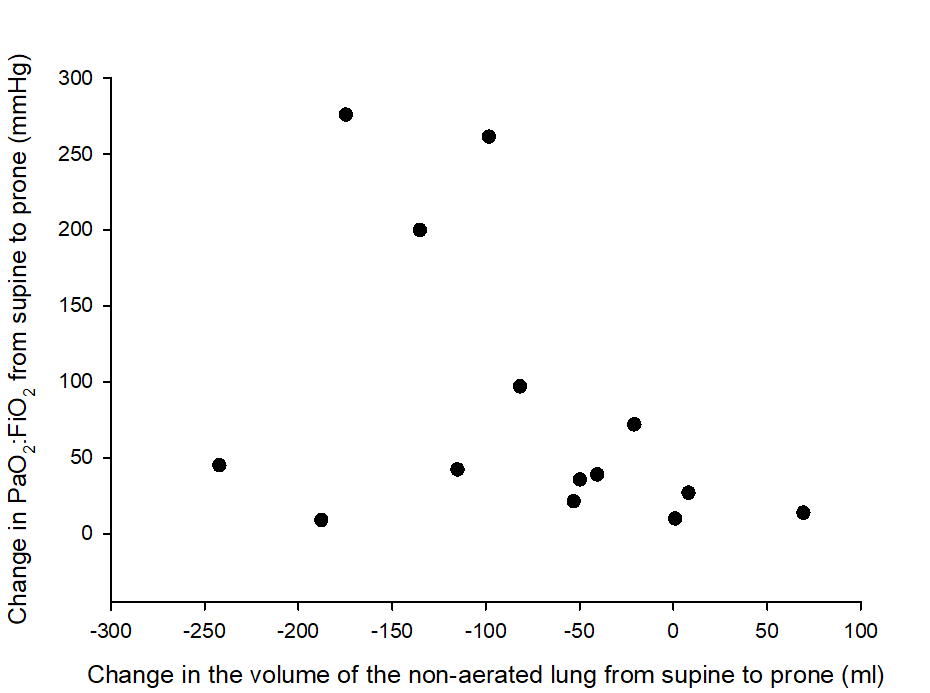


Fourteen mechanically-ventilated patients with COVID-19 were studied in the supine and prone position. Herein we describe the association between the change in the volume of the non-aerated lung, measured with computed tomography, and the change in the arterial tension (PaO_2_) to inspiratory fraction (FiO_2_) of oxygen. A negative change (or decrease) in the volume of the non-aerated lung signals alveolar recruitment. The association was neither strong nor statistically significant (rho -0.415, p=0.134)

**Figure S7.** Impact of prone positioning on alveolar hyperinflation.


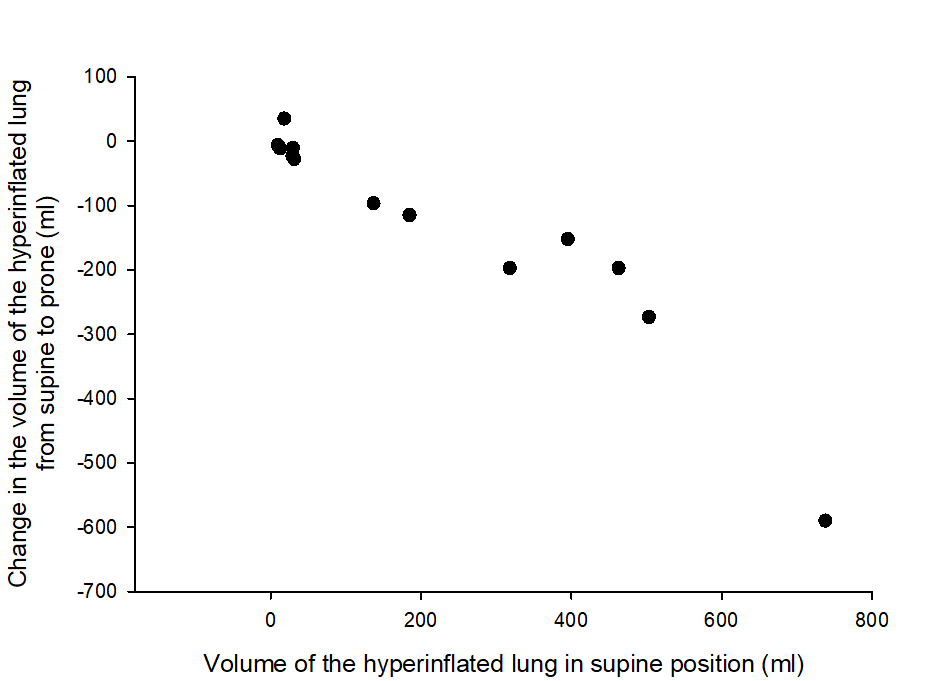


Fifteen patients with COVID-19 requiring mechanical ventilation underwent a lung computed tomography within 2 (1-2) days after endotracheal intubation. Herein we show the association between the change in the volume of the over-aerated compartment induced by prone positioning and the volume of that same compartment in the supine position (rho -0.961, p<0.001).

**Figure S8.** Change in compliance and arterial carbon dioxide tension (PaCO_2_) in response to prone positioning.


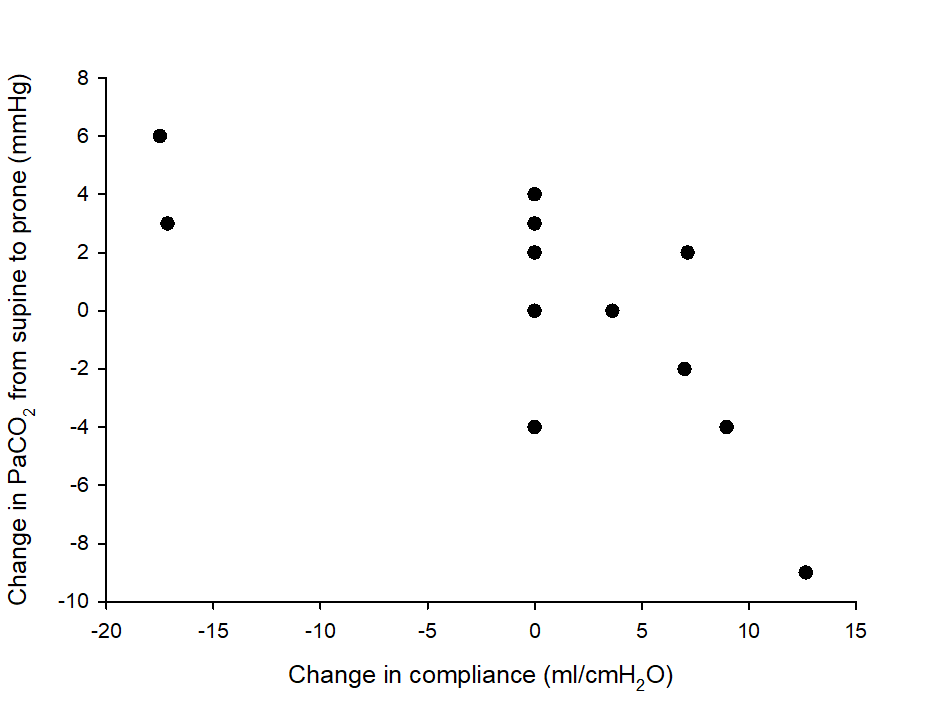


Fourteen mechanically-ventilated patients with COVID-19 were evaluated in the supine and prone position. Herein we show the association between the change in the respiratory system compliance and PaCO_2_ for the same minute ventilation in response to prone positioning (rho -0.713, p=0.005). Data refer to thirteen patients as minute ventilation was changed during prone positioning in one.

**Table S6.** Comparison between patients with less or more hyperinflation at the lung computed tomography (CT).

| **Variable** | **With less**  **hyperinflation** | **With more**  **hyperinflation** | **P-value** |
| --- | --- | --- | --- |
| N | 8 | 7 |  |
| *General characteristics* | | | |
| Males (n [%]) | 4 (50) | 7 (100) | 0.077 |
| Age (years) | 70 (66-72) | 69 (62-75) | 0.867 |
| Body mass index (BMI) (kg/m^2^) | 30 (28-36) | 25 (22-29) | 0.054 |
| Current smoker (n [%]) | 1 (13) | 1 (14) | >0.999 |
| History of COPD (n [%]) | 0 (0) | 0 (0) | >0.999 |
| Time from hospital admission to CT (days) | 4 (3-5) | 5 (3-7) | 0.463 |
| Time from intubation to CT (days) | 2 (1-2) | 1 (1-2) | 0.613 |
| *Ventilatory setting* | | | |
| Tidal volume (ml) | 400 (360-420) | 400 (385-430) | 0.536 |
| Tidal volume (ml/kg of PBW) | 6.8 (6.0-7.0) | 6.1 (5.7-6.5) | 0.189 |
| Respiratory rate (bpm) | 19 (16-21) | 20 (17-22) | 0.779 |
| PEEP (cmH_2_O) | 12 (10-14) | 12 (11-14) | 0.867 |
| FiO_2_ (%) | 0.9 (0.7-1.0) | 1.0 (0.7-1.0) | 0.613 |
| Minute ventilation (L/min) | 7.8 (5.8-8.8) | 7.6 (6.6-9.0) | 0.694 |
| *Respiratory system mechanics* | | | |
| Plateau airway pressure (cmH_2_O) | 20 (20-24) | 23 (21-23) | 0.613 |
| Driving airway pressure (cmH_2_O) | 8 (7-11) | 8 (8-11) | 0.694 |
| Compliance (ml/cmH_2_O) | 44 (36-60) | 50 (38-56) | 0.955 |
| *Gas exchange* | | | |
| Arterial pH | 7.38 (7.34-7.39) | 7.34 (7.25-7.35) | 0.094 |
| PaCO_2_ (mmHg) | 54 (51-59) | 59 (56-60) | 0.121 |
| PaO_2_ (mmHg) | 70 (59-139) | 107 (79-159) | 0.189 |
| PaO_2_/FiO_2_ (mmHg) | 95 (80-167) | 107 (92-179) | 0.152 |
| *Outcome* | | | |
| ICU length of stay (days) | 20 (12-34) | 17 (10-44) | 0.955 |
| Mortality in ICU (n [%]) | 2 (25) | 4 (57) | 0.315 |

Patients were divided in two groups based on the volume of their over-aerated lung measured with CT: ≤31 ml (less hyperinflation) or >31 ml (more hyperinflation). Thirty-one ml was the median value in the overall study population. The resulting volume of the over-aerated lung was 24 (12-30) and 395 (218-493) ml. Herein we compare these two groups for their general characteristics, respiratory system mechanics, and gas exchange. BMI: body mass index. PBW: predicted body weight. PEEP: positive end-expiratory pressure. FiO_2_: inspiratory fraction of oxygen. PaCO_2_: arterial tension of carbon dioxide. PaO_2_: arterial tension of oxygen. The driving airway pressure was the difference between the plateau airway pressure and total PEEP measured with a 5-second end-inspiratory and end-expiratory pause. The compliance was the ratio of the tidal volume to the driving airway pressure. Data are reported as median (Q1-Q3) or proportion. P-value refers to the Mann-Whitney rank-sum test or the Fisher’s exact test.

**References**

E1) Cressoni M, Gallazzi E, Chiurazzi C, et al. Limits of normality of quantitative thoracic CT analysis. Crit Care. 2013;17(3):R93

E2) Pelosi P, D’Andrea L, Vitale G, Pesenti A, Gattinoni L. Vertical gradient of regional lung inflation in adult respiratory distress syndrome. Am J Respir Crit Care Med. 1994;149(1):8-13.
